# Supplementary material for: Comprehensive Annotation and Functional Exploration of MicroRNAs in Lettuce
Source: Front Plant Sci. 2021 Dec 24;12:781836. doi: 10.3389/fpls.2021.781836 (PMC8739914; doi:10.3389/fpls.2021.781836)
Supplement: Supplementary file 3 [file Data_Sheet_9.ZIP › GSM2230753.plot/Lsa-miR159b_Lsat_1_v5_gn_1_64661.1_652_TPlot.pdf]

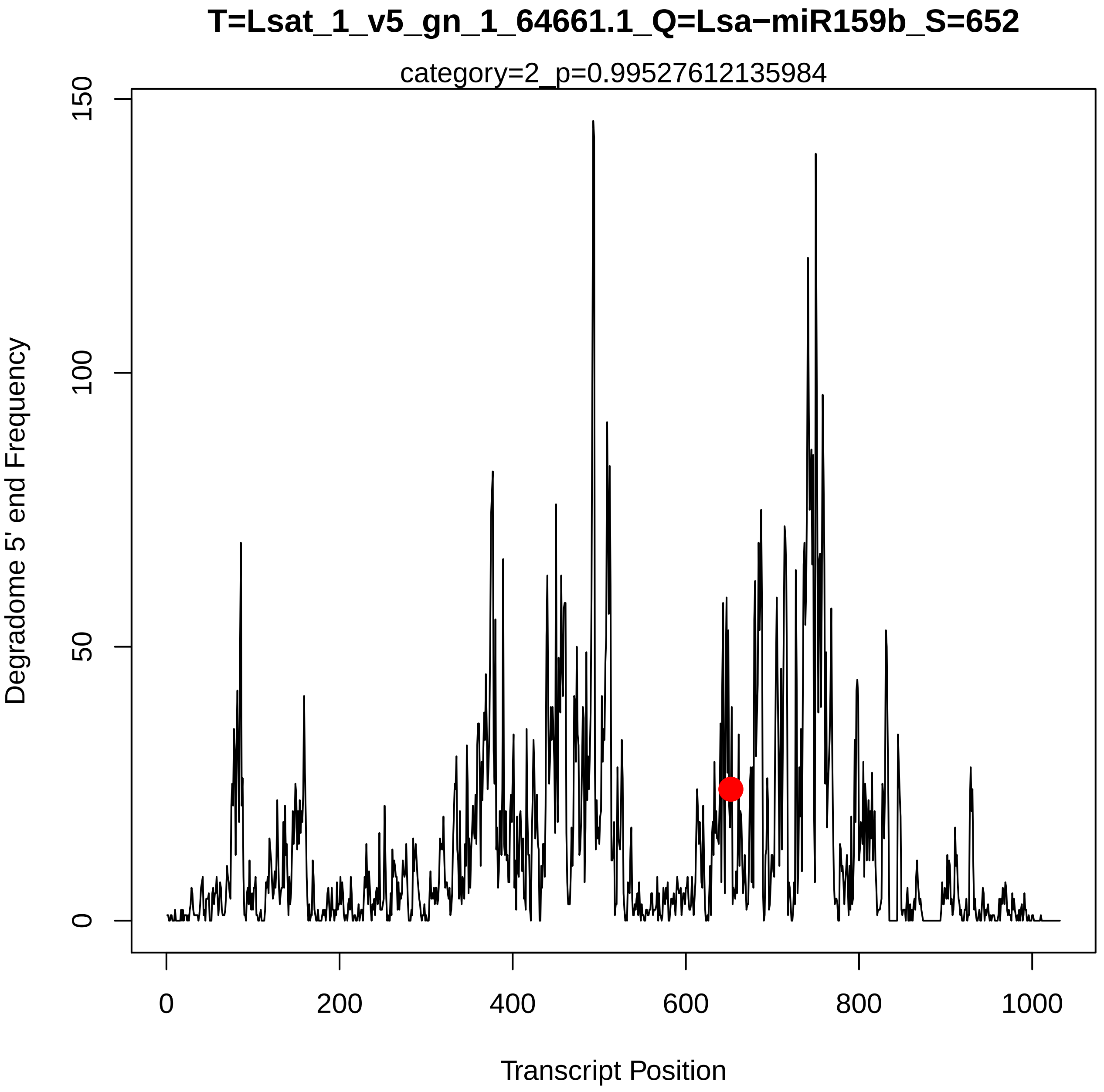
T=Lsat_1_v5_gn_1_64661.1_Q=Lsa−miR159b_S=652
category=2_p=0.99527612135984
Degradome 5' end Frequency
0
50
100
150
0
200
400
600
800
1000
Transcript Position
